# A pilot study for decellularizing porcine cornea for future use in corneal regeneration

**DOI:** 10.1371/journal.pone.0339462

**Published:** 2025-12-31

**Authors:** Natasha Josifovska, Essi M. Niemi, Murugan Ramalingam, Geetha Manivasagam, Hanne Scholz, Goran Petrovski

**Affiliations:** 1 Center for Eye Research and Innovative Diagnostics, Department of Ophthalmology, Oslo University Hospital, and Institute for Clinical Medicine, Faculty of Medicine, University of Oslo, Oslo, Norway; 2 Vascular Biology and Surgery Group, Institute for Surgical Research and Department of Vascular Surgery, Oslo University Hospital, Oslo, Norway; 3 Hybrid Technology Hub, Centre of Excellence, Institute of Basic Medical Sciences, University of Oslo, Oslo, Norway; 4 Centre for Biomaterials, Cellular and Molecular Theranostics, Vellore Institute of Technology, Vellore, Tamil Nadu, India; 5 NanoBioCel Group, Department of Pharmacy and Food Sciences, Faculty of Pharmacy, University of the Basque Country (UPV/EHU), Vitoria-Gasteiz, Spain; 6 Bioaraba Health Research Institute, Jose Atxotegi, s/n, Vitoria-Gasteiz, Spain; 7 Biomedical Research Networking Centre in Bioengineering, Biomaterials and Nanomedicine (CIBER-BBN), Institute of Health Carlos III, Madrid, Spain; 8 Joint Research Laboratory (JRL) on Bioprinting and Advanced Pharma Development, A Joint Venture of TECNALIA (Basque Research and Technology Alliance), Lascaray Research Center, University of the Basque Country (UPV/EHU), Vitoria-Gasteiz, Spain; 9 IKERBASQUE, Basque Foundation for Science, Bilbao, Spain; 10 Institute for Surgical Research and Section for Transplant Surgery, Cell Transplantation and Tissue Engineering Group, Oslo University Hospital, Oslo, Norway; 11 Department of Ophthalmology, University Hospital Split; School of Medicine, University of Split, Split, Croatia; 12 UKLONetwork, University St. Kliment Ohridski, Bitola, North Macedonia; University College London Institute of Child Health, UNITED KINGDOM OF GREAT BRITAIN AND NORTHERN IRELAND

## Abstract

Porcine corneas were decellularized for future use in corneal regeneration by using various washing steps with 3-[(3-cholamidopropyl) dimethylammonio]-1-propanesulfonate (CHAPS) detergent, Benzonase, and Ethylenediaminetetraacetic acid (EDTA). The quality of the decellularized corneas was assessed by quantitative and qualitative measurement of DNA content, glycosaminoglycans (GAGs), immunofluorescent staining for Collagen (Col) I, V, Keratocan, Fibronectin, Laminin, Lumican and Decorin, and Transmission Electron Microscopy (TEM) to observe the structure of the collagen fibrils was used. The decellularization process showed 99.5% DNA content reduction in the corneas and a similar pattern was observed in the preservation of GAGs. Hematoxylin & Eosin (H&E) and immunofluorescent staining showed no presence of cell nuclei, while Alcian blue staining confirmed the presence of GAGs. Col I, V, Keratocan, Fibronectin, Laminin, Lumican and Decorin were still present in the decellularized corneas and TEM microscopy further confirmed the similar patterns of the collagen fibrils in the decellularized, compared to the native corneas. This pilot study showed our method is effective in decellularizing porcine corneas, with a very high amount of DNA being removed, while the GAGs being preserved to an acceptable extent, and the structure and pattern of the collagen fibrils maintained.

## Introduction

Corneal damage often leads to irreversible opacity of the cornea which causes blindness that affects millions of people worldwide [1]. Although many techniques have been employed for cornea transplantation and proven to be effective [2–4], there is a lack of corneal donor tissue for transplantation. Recently, it was found that one cornea is available for every 70 needed, therefore, developing corneal tissue substitutes is of enormous value [5].

Currently, there are several alternative ways to treat corneal opacities, including the use of keratoprosthesis and tissue-engineered corneas. While keratoprosthesis is not commonly used since it does not support tissue regeneration, more emphasis is given to the development of tissue-engineered corneas using different materials such as collagen, fibrin, agarose, silk fibroin, extracellular matrix-derived hydrogels or different synthetic polymers with each of them having its advantages and limitations [6–8]. Another alternative is the use of decellularization methods for obtaining decellularized corneal scaffolds for transplantation.

Tissue for decellularization can be supplied from many species. The most commonly used comes from human donors with otherwise poor quality and not suitable for transplantation due to low endothelial cell density [9–13]. A variety of chemical and physical methods have been established for the decellularization of cornea in recent years [14].

The main purpose of an effective decellularization process is the complete removal of cells from organs or tissues, leaving a cell-free scaffold that consists of its own extracellular matrix (ECM) [15,16].

Currently, there is no optimal technique for decellularization of the cornea, however, a successful procedure should be able to remove exogenous cells and debris, DNA and RNA, and mitochondria, while minimally disrupting the ECM [15,16]. Simple procedures involving fewer steps and minimal usage of chemical/reagents are preferred. A few things are very important including the maintaining of the tissue architecture, proteins, and GAGs, since it is the structure that is responsible for the corneal transparency and function [17,18].

A new blood vessel decellularization technique was successfully developed in our lab and applied to porcine corneas in this pilot study which involves multiple steps using different reagents. Our methods removed DNA to a high extent, while preserving the GAGs to an acceptable extent. Col I, V, Keratocan, Fibronectin, Laminin, Lumican and Decorin could be seen in the decellularized corneas when compared to the native ones. Similar patterns of the collagen fibrils in the decellularized compared to the native corneas could also be confirmed.

## Materials and methods

### Materials

#### Decellularization process.

The porcine eyes were obtained from a local slaughterhouse within 24 h from death and corneas were isolated.

The cornea samples were decellularized with a modified technique for a blood vessel decellularization successfully developed in our lab. The corneas were either kept as a whole or cut half for the decellularization. The corneas were washed in a series of reagents in Nalgene Straight-Sided Wide-Mouth Polycarbonate Jars 500 mL (Thermo Fisher Scientific, Waltham, MA, USA) with continuous shaking in a buffer volume of 200 mL in a New Brunswick Scientific Innova 42 Incubator Shaker (New Brunswick Scientific Co., Inc., Edison, NJ, USA). Additionally, all reagents were supplemented with 5% Dextran (Carl Roth GmbH + Co. KG, Karlsruhe, Germany) with an aim to inhibit corneal tissue swelling during the decellularization steps.

The corneas were first washed in DPBS (Lonza Group AG, Basel, Switzerland) for overnight (o/n) at +4 °C and 80 rpm, followed by a wash with 100 mM EDTA (Sigma-Aldrich, St. Louis, MO, USA) in DPBS, (pH 7.8) for 22 h in +4 °C and 110 rpm. The next step was a detergent wash with 8 mM CHAPS (Abcam, Cambridge, UK) buffer supplemented with 1 M NaCl and 25 mM EDTA in DPBS (pH 7.6) for 22 h at +37 °C and 110 rpm and afterwards incubated for o/n in DPBS at +4 °C and 80 rpm to remove detergent residues from the tissues. Next, the corneas were incubated (2x3 h) with 10 U/mL Benzonase Nuclease (Sigma-Aldrich, St. Louis, MO, USA) supplemented with 1 mM MgCl_2_ in 50 mM Tris buffer (Sigma-Aldrich, St. Louis, MO, USA), at pH 8.1 and +37 °C and 100 rpm, in 100 mL volume sample jars with tissue to buffer ratio of 10 mg wet weight to 1 mL of Benzonase buffer approximately. After the Benzonase washes, the corneas were moved to 2 M NaCl (Sigma-Aldrich, St. Louis, MO, USA) in DPBS hypertonic buffer to wash out residual DNA from the tissue for o/n at +4 °C and 80 rpm. Lastly, the decellularized samples were washed for 2 days in DPBS supplemented with 1 x Gibco Antibiotic-Antimycotic (Thermo Fisher Scientific, Waltham, MA, USA) at +4 °C and 80 rpm, to wash off possible residues from the decellularization reagents. The washing reagents were changed daily.

### Histological evaluation

Part of the corneas was fixed in 10% formalin (Chemi-Teknik AS, Oslo, Norway) for 24-48 h at room temperature (RT). After fixing, the corneas were incubated in a series of 15%, 30% sucrose (Sigma-Aldrich, St. Louis, MO, USA) and lastly, 1:1 30% sucrose and optimal cutting temperature (O.C.T) medium Tissue-Tek OCT Compound (Sakura Finetek Europe B.V., Leiden, The Netherlands) o/n at +4 °C. Lastly, they were embedded in a pure O.C.T. medium and snap-frozen in liquid nitrogen. The frozen blocks were mounted in a cryostat (Leica, Wetzlar, Germany) and 20 µm sections were cut and transferred to SuperFrost slides (Thermo Fisher Scientific, Waltham, MA, USA). Prepared sections were air-dried for up to 1 h at RT and stored at −20 °C for further analysis.

Cryopreserved samples were stained with H&E (Histolab Products AB, Gothenburg, Sweden) to visualize nuclei (10 min staining in Hematoxylin, followed with washing and staining with eosin for additional 10 min), while Alcian blue (Sigma-Aldrich, St. Louis, MO, USA) with Nuclear fast red solution (Sigma-Aldrich, St. Louis, MO, USA) was used to visualize the amount of GAGs preserved. The samples were stained in Alcian blue solution for 30 min, washed in running tap water, and rinsed in distilled water, followed by counterstaining in nuclear fast red solution for 5 min. After washing in running tap water samples were dehydrated through 95% alcohol, and proceeded with 2 changes of absolute alcohol, 3 min each, cleared in xylene and mounted with resinous mounting medium.

### DNA removal and quantification

The samples were stained for the presence of nuclei. Vector TrueVIEW Autofluorescence Quenching Kit (Vector Laboratories, Newark, CA, USA) was used before staining with DAPI (Thermo Fisher Scientific, Waltham, MA, USA). Vector TrueVIEW reagent was added to cover the tissue section and incubated for 2–5 min. Next samples were washed in PBS buffer for 5 min. DAPI staining was followed for 10 min and 2x5 min washing was done before the samples were mounted.

### DNA quantification

To quantify residual DNA in the decellularized corneal tissue, and compared to native, 15–20 mg (wet weight) pieces were cut from 1–3 different sites of each cornea depending on the size of the cornea samples. The pieces were dried with Thermo Scientific Savant SPD131DDA SpeedVac Concentrator (Thermo Fisher Scientific, Waltham, MA, USA), in 70 °C, for 2 h, and the dry weight of the samples was noted down. The samples were processed with DNeasy Blood & Tissue Kit (Qiagen, Hilden, Germany) according to the manufacturer’s instructions, starting by first rehydrating the dried samples to the kit’s ATL buffer and lysed with proteinase K solution. The samples were incubated at 56°C until the tissue pieces were completely lysed, vortexed every 30 min to enhance the lysis. After complete lysis, the samples were further processed and purified according to the manufacturer’s instructions. The purified DNA samples were quantified with Quant-iT PicoGreen dsDNA Assay Kit (Thermo Fisher Scientific, Waltham, MA, USA) according to the manufacturer’s instructions. The samples were excited at 485 nm and the fluorescence emission intensity was measured at 520 nm by using POLARstar Omega (BMG LABTECH GmbH, Ortenberg, Germany) plate reader, all samples were measured in duplicates.

### Evaluation of the ECM

#### GAGs content and quantification.

To quantify the amount of GAGs, 20–25 mg (wet weight) tissue pieces were cut from 1–3 different sites of each cornea depending on the size of the cornea samples from the native and decellularized corneas. The samples were dried with SpeedVac, in 50 °C, for 3 h, and the dry weights of the samples were noted down. GAGs quantification was performed with Blyscan sGAG Assay (Biocolor Ltd., Carrickfergus, UK) by first rehydrating the samples to the 0.2 M sodium phosphate buffer, pH 6.4, introduced in the assay manual. The samples were then digested in papain extraction buffer (Sigma-Aldrich, St. Louis, MO, USA) at 65 °C for 3 h as described in the manual and further processed for the assay according to the manufacturer’s instructions. The absorbance was measured from the samples in duplicates at 650 nm with Victor X5 (PerkinElmer, Waltham, MA, USA) plate reader.

#### Immunofluorescence staining.

Before immunostaining cryosections were kept at RT for 20 min and rehydrated with DPBS (Life Technologies Europe B.V., Bleiswijk, The Netherlands) 3x5 min. Permeabilization was done by using 0.1% Triton X-100 (Sigma Life Science (Sigma-Aldrich), St. Louis, MO, USA) in DPBS for 10 min followed by 3x5 min washing with DPBS. Unspecific binding sites were blocked using 5% BSA (Sigma Life Science (Sigma-Aldrich), St. Louis, MO, USA) in DPBS for 1 h. Primary antibodies Col I, Col V (Abcam, Cambridge, UK) and Keratocan (Bioss Antibodies, Woburn, MA, USA), Fibronectin, Laminin, Lumican, Decorin (Invitrogen, Thermo Fisher Scientific, Waltham, MA, USA) (detailed list of antibodies is provided in (S1 Table), Supporting Information) were prepared in 0.5% BSA and samples were incubated for o/n at 4°C. The next steps included washing with DPBS and 1 h incubation at RT with secondary antibody Alexa fluor 488 (Invitrogen, Thermo Fisher Scientific, Waltham, MA, USA), protected from light. Counter-staining was performed with DAPI (Thermo Fisher Scientific, Waltham, MA, USA) following the manufacturer’s instructions and mounting was done in a single step using the Prolong diamond solution (Invitrogen, Thermo Fisher Scientific, Waltham, MA, USA). Results were visualized and recorded using Zeiss fluorescent microscope.

### TEM

TEM was performed to analyze the ultrastructure of the cornea and the alignment of the collagen fibrils and structure.

The primary fixation of the samples was with 2.0% Glutaraldehyde in Cacodylate‐buffer (Chemi-Teknik AS, Oslo, Norway) (pH 7.4) o/n at 4 °C. Next, samples were washed with 0.2M of cold Cacodylate buffer, 4x15 min and postfixed with 1% Osmium tetroxide/OSO4 (Chemi-Teknik AS, Oslo, Norway) in 0.2M Cacodylate-buffer for 30–40 min, followed by 3x15 min washing steps with 0.2M Cacodylate-buffer. The fixed samples were dehydrated by incubation in a series of alcohol by increasing its concentration gradually. Following dehydration, embedding was performed in Propylenoxid-2x5 min, with 1:1 mix of Epon+Propylenoxid for 60 min, and 100% Epon mix (Sigma-Aldrich, St. Louis, MO, USA) for o/n. Next, samples were embedded in an Epon mix and polymerized at 60–70°C for 48 h.

A Leica Ultracut Ultramicrotome was used to cut ultra-thin (60–70 nm) sections and contrast was enhanced with uranyl acetate and lead cytrate. The images were taken by a Tecnai 12 Transmission Electron Microscope (Phillips, Amsterdam, the Netherlands).

### Statistical analysis

All data are presented as mean values ± standard deviations. Nonparametric Mann-Whitney U test was used to determine statistically significant differences (GraphPad Prism 5, GraphPad Software, Inc., San Diego, CA, USA). Statistical significance was set at p-value < 0.05.

### Biological tissues and ethics statements

Porcine eyes were obtained from a local slaughterhouse within 24 h from death.

## Results

**Fig 1:**
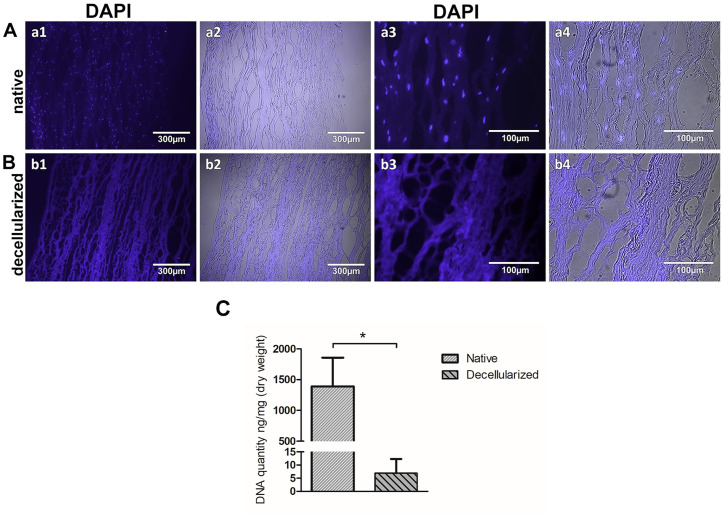
Nuclear staining of native and decellularized corneas. Native Aa1 DAPI blue (10x), Aa2 merged BF+DAPI (10x), Aa3 DAPI blue (40x), Aa4 merged BF+DAPI (40x), and decellularized Bb1 DAPI blue (10x), Bb2 merged BF+DAPI (10x), Bb3 DAPI blue (40x), Bb4 merged BF+DAPI (40x) corneal stroma. DNA quantity in ng/mg in native vs decellularized cornea n = 4 **(C)**. Data shown are mean ± standard deviation (SD).

**Fig 2:**
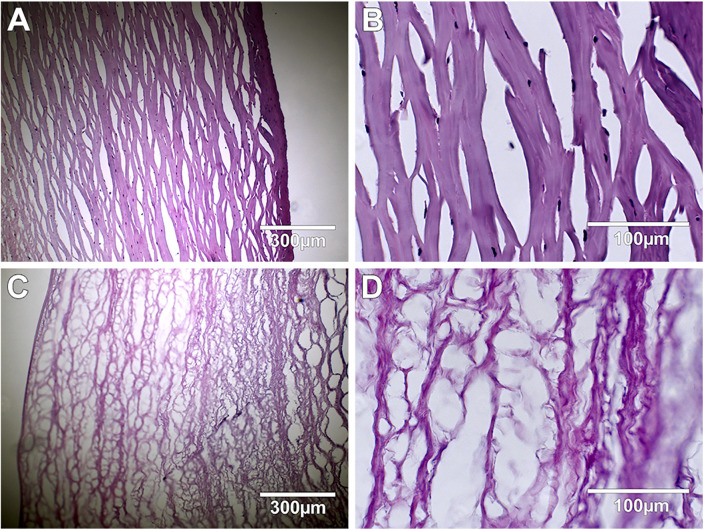
H&E staining of native and decellularized corneas. Native A (10x), B (40x), and decellularized C (10x), D (40x) corneal stroma.

The decellularization process showed 99.5% DNA content reduction in the corneas when the decellularization reagents were supplemented with 5% Dextran; this was further confirmed by immunofluorescent (Fig 1), and H&E staining which showed the presence of nuclei in the native corneas (Fig 2 A, B), being absent in the decellularized samples (Fig 2 C, D).

**Fig 3:**
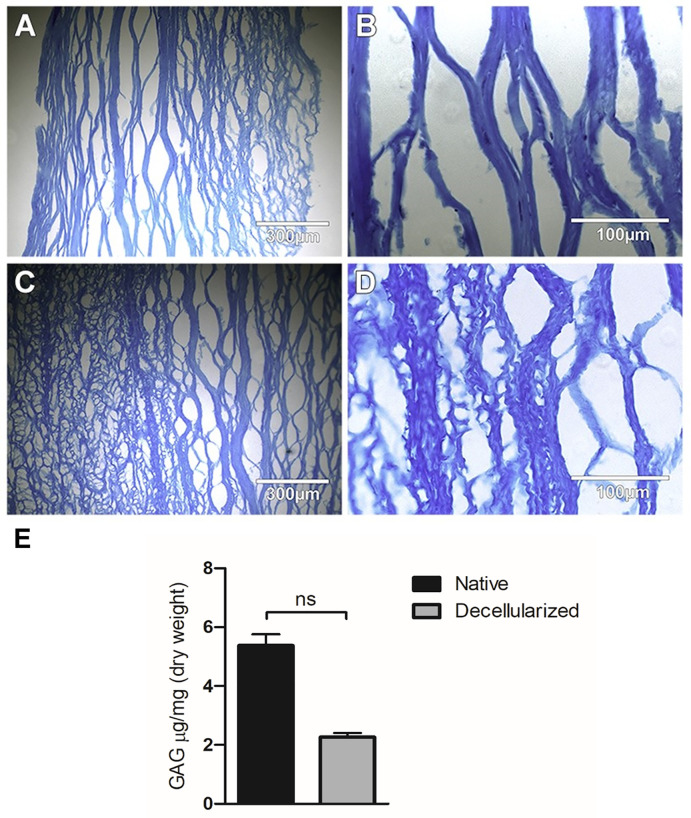
Alcian blue staining of native and decellularized corneas. Native A (10x), B (40x), and decellularized C (10x), D (40x) corneal stroma. GAGs quantity preserved in ng/mg in native vs decellularized cornea n = 4 **(E)**. Data shown are mean ± standard deviation (SD).

Similar pattern (Fig 3) was observed in the preservation of GAGs, with 42.13%. In addition, Alcian blue staining qualitatively confirmed the presence of GAGs in the native corneas (Fig 3 A, B), which were further preserved in the decellularized ones (Fig 3 C, D).

**Fig 4:**
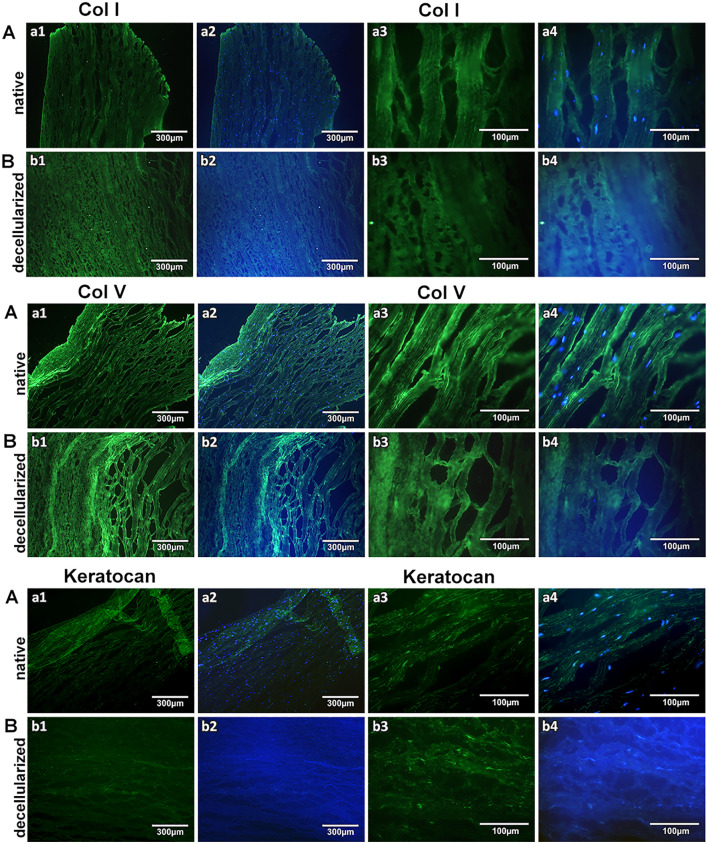
Col I, V and Keratocan staining of native and decellularized corneas. Native Aa1 green (10x), Aa2 merged Col I/Col V/Keratocan+DAPI (10x), Aa3 green (40x), Aa4 merged Col I/Col V/Keratocan+DAPI (40x), and decellularized Bb1 green (10x), Bb2 merged Col I/Col V/Keratocan+DAPI (10x), Bb3 green (40x), Bb4 merged Col I/Col V/Keratocan+DAPI (40x) corneal stroma.

Furthermore, Col I, V and Keratocan (Fig 4) were still present in the decellularized corneas when compared to the native ones.

**Fig 5:**
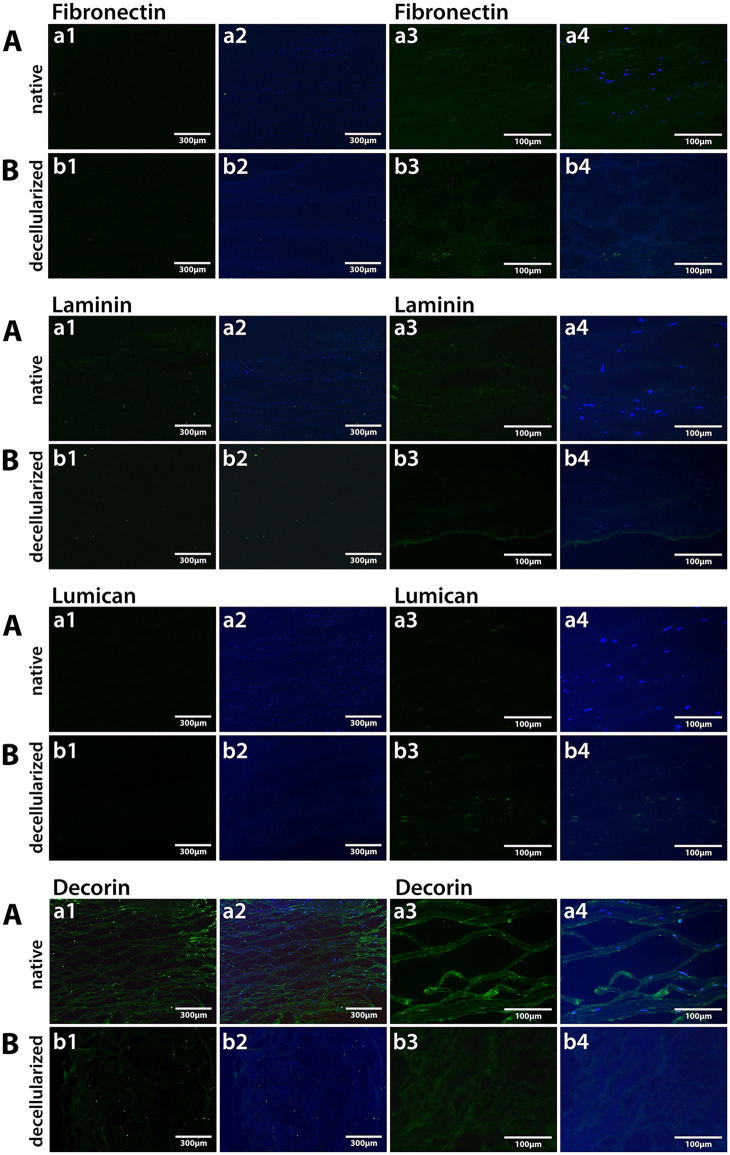
Fibronectin, Laminin, Lumican and Decorin staining of native and decellularized corneas. Native Aa1 green (10x), Aa2 merged Fibronectin/Laminin/Lumican/Decorin+DAPI (10x), Aa3 green (40x), Aa4 merged Fibronectin/Laminin/Lumican/Decorin +DAPI (40x), and decellularized Bb1 green (10x), Bb2 merged Fibronectin/Laminin/Lumican/Decorin +DAPI (10x), Bb3 green (40x), Bb4 merged Fibronectin/Laminin/Lumican/Decorin +DAPI (40x) corneal stroma.

Fibronectin, Laminin and Lumican (Fig 5) were lightly present in both native and decellularized corneas, whereas decorin showed stronger staining.

**Fig 6:**
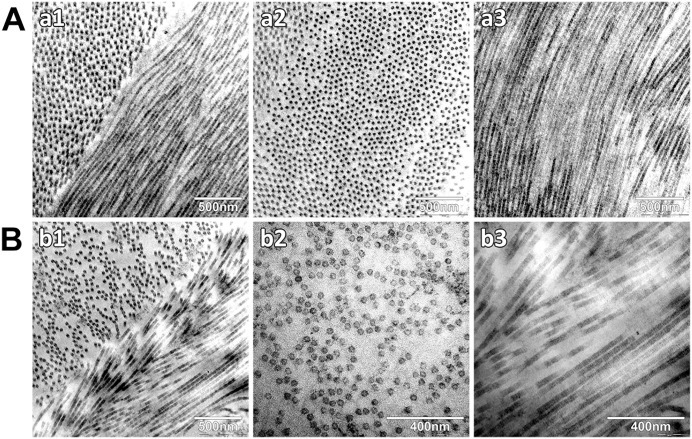
Transmission electron microscopy pictures of Collagen fibril organization. Native A (a1, a2, a3) and decellularized B (b1, b2, b3) corneal stroma (scale 400 and 500 nm).

TEM microscopy further confirmed the similar patterns of the collagen fibrils in the decellularized (Fig 6 B), compared to the native- corneas (Fig 6 A). In the pictures, we can see the collagen fibril organization in native corneal stroma compared to decellularized one.

## Discussion

Despite being the most successful organ transplant operation, corneal transplantation is accompanied with two major complications: immunological rejection and donor-derived infections [11,19]. Although nowadays there exist several donor tissue alternatives to overcome difficulties concerning biocompatibility and immunoreaction of the host towards engineered tissue, decellularized tissues appear capable of reducing such issues. Discarded tissue from myopia correction procedures or donated corneas unsuitable for transplantation can be used as a potential source for decellularized corneal tissue production [12,20].

Some known methods for decellularization results in incomplete cell removal [21,22], while other are not evaluated completely. In addition, there exists no optimal technique for corneal decellularization, while different research groups have used similar protocols and gained different results [1,10,16].

The decellularization techniques can be classified into three types: physical, chemical, and biological. While the physical methods such as freeze-thaw appear safe, the ECM in such procedures seems disrupted, thus resulting in lower corneal transparency. Chemical agents have been the most used way for decellularizing the cornea, among them being ionic detergents like sodium dodecyl sulfate (SDS) non-ionic detergents like Triton X-100, and zwitterionic detergents [16,17,23]. From biological agents, enzymes such as Trypsin and Dispase II have been used for lysing the corneal cells.

Several steps need to be verified after the decellularization process. The most important is the detection of cells and cellular components such as the presence of DNA. Such remnants can lead to inflammatory or immunological reactions and rejection of the transplanted construct [14]. For this reason, the amount of DNA should be quantified by using DNA based assays comparing the cornea before and after the decellularization process. Another part of this verification step is the identification of presence of cells by using histology-based methods like H&E and DAPI staining to fluorescently identify cell nuclei.

Our protocol was designed to achieve effective decellularization while preserving key structural and biochemical components of the cornea, including collagen, ECM, and GAGs, with minimal residual DNA. This balance is critical because the cornea’s optical clarity and mechanical integrity depend on maintaining its native architecture and composition. Rigorous protocols that completely eliminate cellular debris often compromise ECM integrity, leading to opacities and impaired function [24,25]. To address this challenge, we selected CHAPS as the primary detergent due to its zwitterionic nature, which offers efficient cell lysis with reduced cytotoxicity compared to ionic detergents. CHAPS has been successfully applied to thinner tissues such as lung and vascular grafts [26]. However, its milder action can leave residual cellular fragments, which represents a limitation of this approach. To mitigate this, we incorporated sequential steps designed to enhance DNA removal while minimizing structural damage. The protocol begins with EDTA treatment to disrupt cell–cell and cell–matrix adhesions, facilitating subsequent detergent penetration. CHAPS was then applied in hypertonic buffer to solubilize membranes and nuclear material, with low EDTA concentration included to inhibit protease activity [27]. To ensure efficient nucleic acid clearance, two benzonase washes were performed, followed by a hypertonic NaCl wash to remove residual debris. All steps were optimized for temperature and pH to balance enzymatic activity and tissue preservation, and gentle agitation was used to improve reagent-tissue contact without mechanical damage. The main advantage of this approach lies in its ability to retain ECM components-particularly collagen and GAGs while achieving very low residual DNA levels, as confirmed by our results and consistent with previous findings [28]. This preservation is essential for maintaining corneal transparency and mechanical strength, and for creating a biologically favorable scaffold for recellularization. However, the limitation remains that some cytoplasmic remnants may persist, which could theoretically elicit immune responses. Nonetheless, literature suggests that partial retention of ECM and minimal debris may reduce host inflammatory reactions compared to overly aggressive protocols that damage structural proteins [26].

In some studies, DNA has been detected only by H&E or DAPI staining [11,14,29], which is not sufficient to show the DNA removal. While these studies have shown an average DNA reduction of 97%, which seems to be incomplete [21,22,29], we hereby show that the decellularization process could remove 99.5% of the DNA content in the porcine corneas, by quantifying the DNA using Quant-iT PicoGreen dsDNA Assay Kit, and further confirming by H&E and DAPI staining. Corneal tissue swells significantly in cell culture conditions due to its high GAGs content [30]. Furthermore, the small leucine-rich proteoglycans (PGs), such as Keratocan and Decorin, play a significant role in collagen fibrillogenesis in terms of collagen assembly nucleation, and linear and lateral fibril development [23]. Therefore, osmoregulatory agents such as dextran and glycerol have been used to restore the natural shape of the collagen fibrils, allowing decorin molecules to restore corneal transparency. Glycerol helps in maintaining tissue hydration, but it also causes severe damage to the corneal ultrastructure due to fast dehydration [17,30]. Dextran has been routinely used before keratoplasty at a concentration of 5% to maintain the hydration state and thickness of the corneas in storage via colloid osmotic pressure [30]. Although dextran has been used in the corneal decellularization process at a concentration of 3.5%, and no rationale has been provided for the use of this specific concentration [29], we have used the 5% concentration during the process.

The next important step after decellularization is verification of the ECM composition, which can be validated by quantifying the amount of Col and GAG present.

Collagens and PGs are the major extracellular components that make up the corneal stroma. In the stroma, Col type I prevails with Col types V, VI, XI, XII, and XIV being also present [14,17]. PGs are proteins that contain GAGs which are negatively charged linear complex molecules required for regulating corneal collagen fibrillar assembly and preserving the stromal structure organization thereby providing corneal hydration, structural integrity, transparency, and thickness [31,32]. The PGs have been also shown to be necessary for maintaining corneal homeostasis, epithelial cell differentiation and wound healing [33]. Keratan sulphate (KS), dermatan sulphate, and chondroitin sulphate are the GAGs found in the corneal stroma [34]. KS is one of the cornea’s most important GAGs. In the cornea, KS-GAGs form complexes with one of three core proteins: Lumican, Keratocan, or Mimecan, and so exist as PGs. Both Lumican and Keratocan are needed for appropriate embryonic cornea development and adult corneal structure maintenance [23,35,36]. Furthermore, Fibronectin and Laminin are essential for maintaining corneal structure and function [37], while Decorin and Lumican influence collagen organization and are relevant to corneal transparency and biomechanics [38].

The histological staining using picro sirius red to identify collagen [15] or Alcian blue to identify the presence of GAGs can quantify the signal intensity and thus determine their change after decellularization [10]. Immunofluorescent staining can be also used to identify specific collagens of interest. GAG content has been shown only by Alcian Blue by others [21] and also in our study, where we quantified the amount of GAG preserved after the decellularization process. On Fig 3, the intensity of the color when the stroma was stained with Alcian blue was same after the decellularization process of the corneas when compared to the native corneas, which indicates unchanged presence of GAGs. Furthermore, Col I, V and Keratocan appear preserved after the decellularization process as shown in Fig 4. Fibronectin, Laminin and Lumican exhibited low expression levels in the native cornea and were similarly preserved following decellularization, whereas Decorin remained strongly expressed in both native and decellularized corneal tissue as illustrated in Fig 5.

Structural evaluation is of enormous importance, and it is achieved by TEM. The structure of the cornea contributes to the transparency and, therefore, it is crucial the collagen fibrils are properly aligned with regular spacing between them; this is believed to be regulated by PGs, which have been shown in normal corneas to form ring-like structures around collagen fibrils [39,40]. Here, we show that the structure and alignment of the collagen fibrils was preserved after the decellularization process.

Benzonase is a genetically engineered endonuclease used as a decellularizing enzyme. Due to its low molecular weight, it can quickly enter the stroma, removing cells from the ECM, and being easily washed out thus not damaging the collagen fibrils [41,42]. Benzonase can degrade all forms of DNA and RNA in a wide range of pH values and temperatures. Endonucleases such as benzonase may more effectively remove DNA because they cleave nucleotides mid-sequence, while exonucleases cleave nucleotides from the ends of the DNA molecule [43,44].

CHAPS is mainly used to decellularize thin tissues since it is not good as a permeating agent [45]. When CHAPS was used to decellularize human amniotic membrane matrix for the purpose of cardiac regeneration, GAGs were relatively conserved when compared to SDS-driven decellularization [46], the latter been also shown to have toxic effects on the ECM after decellularization of porcine pericardium [47]. Furthermore, when using CHAPS, it is also important to take its pH into consideration. CHAPS has been tested in a pH range from 8 to 12 when decellularizing lungs, and has been found to preserve GAGs to a greater extent when the pH was in a more neutral range [26]. The use of CHAPS for corneal decellularization has been poorly reported, [48], however, in our case, using CHAPS detergent at pH 7.6 was very sufficient in the process of decellularization.

## Conclusion

Our decellularization method demonstrates suitability for porcine corneas, yielding well-preserved tissues with native structure and integrity. These decellularized corneas have potential applications in tissue transplantation, effectively addressing corneal damage needs and extending tissue lifespan. Our protocol prioritizes structural and biochemical integrity over complete cellular clearance, reflecting the principle that successful decellularization is a balance between removing immunogenic material and preserving the native tissue architecture necessary for function and biocompatibility. Furthermore, it offers a versatile approach for tissue engineering, suggesting the feasibility of using animal-sourced decellularized tissues for human constructs. Overall, our findings support the potential of the decellularized cornea as a bioscaffold for corneal regeneration.

## Supporting information

S1 Table List of antibodies used for immunohistochemistry. (DOCX)

S2 File Raw data supporting the analysis. (XLSX)

## References

[1] Dong M, Zhao L, Wang F, Hu X, Li H, Liu T, et al. Rapid porcine corneal decellularization through the use of sodium N-lauroyl glutamate and supernuclease. J Tissue Eng. 2019;10:2041731419875876. doi: 10.1177/2041731419875876 31588337 PMC6740050

[2] Tan DTH, Dart JKG, Holland EJ, Kinoshita S. Corneal transplantation. Lancet. 2012;379(9827):1749–61. doi: 10.1016/S0140-6736(12)60437-1 22559901

[3] Ple-Plakon PA, Shtein RM. Trends in corneal transplantation: indications and techniques. Curr Opin Ophthalmol. 2014;25(4):300–5. doi: 10.1097/ICU.0000000000000080 24865170

[4] Singh R, Gupta N, Vanathi M, Tandon R. Corneal transplantation in the modern era. Indian J Med Res. 2019;150(1):7–22. doi: 10.4103/ijmr.IJMR_141_19 31571625 PMC6798607

[5] Gain P, Jullienne R, He Z, Aldossary M, Acquart S, Cognasse F, et al. Global Survey of Corneal Transplantation and Eye Banking. JAMA Ophthalmol. 2016;134(2):167–73. doi: 10.1001/jamaophthalmol.2015.4776 26633035

[6] Isidan A, Liu S, Chen AM, Zhang W, Li P, Smith LJ, et al. Comparison of porcine corneal decellularization methods and importance of preserving corneal limbus through decellularization. PLoS One. 2021;16(3):e0243682. doi: 10.1371/journal.pone.0243682 33667231 PMC7935270

[7] Lagali N. Corneal Stromal Regeneration: Current Status and Future Therapeutic Potential. Curr Eye Res. 2020;45(3):278–90. doi: 10.1080/02713683.2019.1663874 31537127

[8] Palchesko RN, Carrasquilla SD, Feinberg AW. Natural Biomaterials for Corneal Tissue Engineering, Repair, and Regeneration. Adv Healthc Mater. 2018;7(16):e1701434. doi: 10.1002/adhm.201701434 29845780

[9] da Mata Martins TM, de Carvalho JL, da Silva Cunha P, Gomes DA, de Goes AM. Induction of Corneal Epithelial Differentiation of Induced Pluripotent and Orbital Fat-Derived Stem Cells Seeded on Decellularized Human Corneas. Stem Cell Rev Rep. 2022;18(7):2522–34. doi: 10.1007/s12015-022-10356-6 35247143

[10] Fernández-Pérez J, Madden PW, Brady RT, Nowlan PF, Ahearne M. The effect of prior long-term recellularization with keratocytes of decellularized porcine corneas implanted in a rabbit anterior lamellar keratoplasty model. PLoS One. 2021;16(6):e0245406. doi: 10.1371/journal.pone.0245406 34061862 PMC8168847

[11] Islam MM, Sharifi R, Mamodaly S, Islam R, Nahra D, Abusamra DB, et al. Effects of gamma radiation sterilization on the structural and biological properties of decellularized corneal xenografts. Acta Biomater. 2019;96:330–44. doi: 10.1016/j.actbio.2019.07.002 31284096 PMC7043233

[12] Mertsch S, Hasenzahl M, Reichl S, Geerling G, Schrader S. Decellularized human corneal stromal cell sheet as a novel matrix for ocular surface reconstruction. J Tissue Eng Regen Med. 2020;14(9):1318–32. doi: 10.1002/term.3103 32652796

[13] Porzionato A, Stocco E, Barbon S, Grandi F, Macchi V, De Caro R. Tissue-engineered grafts from human decellularized extracellular matrices: A systematic review and future perspectives. Int J Mol Sci. 2018;19(12). 10.3390/ijms19124117 PMC6321114 30567407

[14] Ahearne M. Corneal extracellular matrix decellularization. Methods Cell Biol. 2020;157:81–95. doi: 10.1016/bs.mcb.2019.10.013 32334721

[15] Fernández-Pérez J, Ahearne M. Decellularization and recellularization of cornea: Progress towards a donor alternative. Methods. 2020;171:86–96. doi: 10.1016/j.ymeth.2019.05.009 31128238

[16] Isidan A, Liu S, Li P, Lashmet M, Smith LJ, Hara H, et al. Decellularization methods for developing porcine corneal xenografts and future perspectives. Xenotransplantation. 2019;26(6):e12564. doi: 10.1111/xen.12564 31659811 PMC6908750

[17] Wilson SL, Sidney LE, Dunphy SE, Dua HS, Hopkinson A. Corneal Decellularization: A Method of Recycling Unsuitable Donor Tissue for Clinical Translation?. Curr Eye Res. 2016;41(6):769–82. doi: 10.3109/02713683.2015.1062114 26397030 PMC4926783

[18] Yoon CH, Choi HJ, Kim MK. Corneal xenotransplantation: Where are we standing?. Prog Retin Eye Res. 2021;80:100876. doi: 10.1016/j.preteyeres.2020.100876 32755676 PMC7396149

[19] El Zarif M, Alió Del Barrio JL, Arnalich-Montiel F, De Miguel MP, Makdissy N, Alió JL. Corneal Stroma Regeneration: New Approach for the Treatment of Cornea Disease. Asia Pac J Ophthalmol (Phila). 2020;9(6):571–9. doi: 10.1097/APO.0000000000000337 33181549

[20] Fernández-Pérez J, Madden PW, Ahearne M. Engineering a Corneal Stromal Equivalent Using a Novel Multilayered Fabrication Assembly Technique. Tissue Eng Part A. 2020;26(19–20):1030–41. doi: 10.1089/ten.TEA.2020.0019 32368948 PMC7580631

[21] da Mata Martins TM, da Silva Cunha P, Rodrigues MA, de Carvalho JL, de Souza JE, de Carvalho Oliveira JA, et al. Epithelial basement membrane of human decellularized cornea as a suitable substrate for differentiation of embryonic stem cells into corneal epithelial-like cells. Mater Sci Eng C Mater Biol Appl. 2020;116:111215. doi: 10.1016/j.msec.2020.111215 32806330

[22] Lin H-J, Wang T-J, Li T-W, Chang Y-Y, Sheu M-T, Huang Y-Y, et al. Development of Decellularized Cornea by Organic Acid Treatment for Corneal Regeneration. Tissue Eng Part A. 2019;25(7–8):652–62. doi: 10.1089/ten.TEA.2018.0162 30244654

[23] Fernández-Pérez J, Ahearne M. The impact of decellularization methods on extracellular matrix derived hydrogels. Sci Rep. 2019;9(1):14933. doi: 10.1038/s41598-019-49575-2 31624357 PMC6797749

[24] Massoudi D, Malecaze F, Galiacy SD. Collagens and proteoglycans of the cornea: importance in transparency and visual disorders. Cell Tissue Res. 2016;363(2):337–49. doi: 10.1007/s00441-015-2233-5 26205093

[25] Xuan M, Wang S, Liu X, He Y, Li Y, Zhang Y. Proteins of the corneal stroma: importance in visual function. Cell Tissue Res. 2016;364(1):9–16. doi: 10.1007/s00441-016-2372-3 26905288

[26] Tsuchiya T, Balestrini JL, Mendez J, Calle EA, Zhao L, Niklason LE. Influence of pH on extracellular matrix preservation during lung decellularization. Tissue Eng Part C Methods. 2014;20(12):1028–36. doi: 10.1089/ten.TEC.2013.0492 24735501 PMC4241865

[27] Simsa R, Padma AM, Heher P, Hellström M, Teuschl A, Jenndahl L, et al. Systematic in vitro comparison of decellularization protocols for blood vessels. PLoS One. 2018;13(12):e0209269. doi: 10.1371/journal.pone.0209269 30557395 PMC6296505

[28] Liu J, Li Z, Li J, Liu Z. Application of benzonase in preparation of decellularized lamellar porcine corneal stroma for lamellar keratoplasty. J Biomed Mater Res A. 2019;107(11):2547–55. doi: 10.1002/jbm.a.36760 31330094 PMC6771539

[29] Lynch AP, Wilson SL, Ahearne M. Dextran preserves native corneal structure during decellularization. Tissue Eng Part C Methods. 2016;22(6):561–72. 27068608 10.1089/ten.TEC.2016.0017

[30] Polisetti N, Schmid A, Schlötzer-Schrehardt U, Maier P, Lang SJ, Steinberg T, et al. A decellularized human corneal scaffold for anterior corneal surface reconstruction. Sci Rep. 2021;11(1):2992. doi: 10.1038/s41598-021-82678-3 33542377 PMC7862698

[31] Pacella E, Pacella F, De Paolis G, Parisella FR, Turchetti P, Anello G, et al. Glycosaminoglycans in the human cornea: age-related changes. Ophthalmology and Eye Diseases. 2015;7:OED.S17204. 10.4137/OED.S17204 PMC4310673 25674020

[32] Hatami-Marbini H. On the Mechanical Roles of Glycosaminoglycans in the Tensile Properties of Porcine Corneal Stroma. Invest Ophthalmol Vis Sci. 2023;64(4):3. doi: 10.1167/iovs.64.4.3 37014650 PMC10080947

[33] Puri S, Coulson-Thomas YM, Gesteira TF, Coulson-Thomas VJ. Distribution and Function of Glycosaminoglycans and Proteoglycans in the Development, Homeostasis and Pathology of the Ocular Surface. Front Cell Dev Biol. 2020;8:731. doi: 10.3389/fcell.2020.00731 32903857 PMC7438910

[34] Bron AJ. The architecture of the corneal stroma. Br J Ophthalmol. 2001;85(4):379–81. doi: 10.1136/bjo.85.4.379 11264120 PMC1723930

[35] Quantock AJ, Young RD, Akama TO. Structural and biochemical aspects of keratan sulphate in the cornea. Cell Mol Life Sci. 2010;67(6):891–906. doi: 10.1007/s00018-009-0228-7 20213925 PMC11115788

[36] Kao WW-Y, Liu C-Y. Roles of lumican and keratocan on corneal transparency. Glycoconj J. 2002;19(4–5):275–85. doi: 10.1023/A:1025396316169 12975606

[37] Gandhi S, Jain S. The anatomy and physiology of cornea. In: Cortina MS, de la Cruz J, editors. Keratoprostheses and artificial corneas: fundamentals and surgical applications. Berlin, Heidelberg: Springer Berlin Heidelberg. 2015. p. 19–25.

[38] Neame PJ, Kay CJ, McQuillan DJ, Beales MP, Hassell JR. Independent modulation of collagen fibrillogenesis by decorin and lumican. Cell Mol Life Sci. 2000;57(5):859–63. doi: 10.1007/s000180050048 10892350 PMC11146782

[39] Meek KM, Knupp C. Corneal structure and transparency. Prog Retin Eye Res. 2015;49:1–16. doi: 10.1016/j.preteyeres.2015.07.001 26145225 PMC4655862

[40] Shah A, Brugnano J, Sun S, Vase A, Orwin E. The development of a tissue-engineered cornea: biomaterials and culture methods. Pediatr Res. 2008;63(5):535–44. doi: 10.1203/PDR.0b013e31816bdf54 18427299

[41] Liu J, Li Z, Li J, Liu Z. Application of benzonase in preparation of decellularized lamellar porcine corneal stroma for lamellar keratoplasty. J Biomed Mater Res A. 2019;107(11):2547–55. doi: 10.1002/jbm.a.36760 31330094 PMC6771539

[42] Moffat D, Ye K, Jin S. Decellularization for the retention of tissue niches. J Tissue Eng. 2022;13:20417314221101151. doi: 10.1177/20417314221101151 35620656 PMC9128068

[43] Crapo PM, Gilbert TW, Badylak SF. An overview of tissue and whole organ decellularization processes. Biomaterials. 2011;32(12):3233–43. doi: 10.1016/j.biomaterials.2011.01.057 21296410 PMC3084613

[44] Hussein KH, Park K-M, Kang K-S, Woo H-M. Biocompatibility evaluation of tissue-engineered decellularized scaffolds for biomedical application. Mater Sci Eng C Mater Biol Appl. 2016;67:766–78. doi: 10.1016/j.msec.2016.05.068 27287176

[45] Mendibil U, Ruiz-Hernandez R. Tissue-specific decellularization methods: rationale and strategies to achieve regenerative compounds. 2020;21(15). 10.3390/ijms21155447 PMC7432490 32751654

[46] Henry JJD, Delrosario L, Fang J. Development of injectable amniotic membrane matrix for postmyocardial infarction tissue repair. J Tissue Eng Regen Med. 2020;9(2):e1900544. 10.1002/adhm.201900544 PMC6986802 31778043

[47] Mallis P, Michalopoulos E, Dimitriou C, Kostomitsopoulos N, Stavropoulos-Giokas C. Histological and biomechanical characterization of decellularized porcine pericardium as a potential scaffold for tissue engineering applications. Biomed Mater Eng. 2017;28(5):477–88. doi: 10.3233/BME-171689 28854488

[48] Marin-Tapia HA, Romero-Salazar L, Arteaga-Arcos JC, Rosales-Ibáñez R, Mayorga-Rojas M. Micro-mechanical properties of corneal scaffolds from two different bio-models obtained by an efficient chemical decellularization. J Mech Behav Biomed Mater. 2021;119:104510. doi: 10.1016/j.jmbbm.2021.104510 33872923

