# Supplementary material for: A pilot study for decellularizing porcine cornea for future use in corneal regeneration
Source: PLoS One. 2025 Dec 31;20(12):e0339462. doi: 10.1371/journal.pone.0339462 (PMC12755736; doi:10.1371/journal.pone.0339462)
Supplement: S1 Table — (DOCX) [file pone.0339462.s001.docx]

***Table S1****. Details of the antibodies used for immunohistochemistry.*

| **Antibody** | **Catalog No.** | **Company** | **Clonality** | **Dilution** |
| --- | --- | --- | --- | --- |
| Collagen I | Ab34710 | Abcam | Rabbit  Polyclonal | 1:200 |
| Collagen V | Ab7046 | Abcam | Rabbit  Polyclonal | 1:200 |
| Keratocan | bs-11054R | Bioss | Rabbit  Polyclonal | 1:200 |
| Fibronectin | PA5-29578 | Invitrogen | Rabbit  Polyclonal | 1:100 |
| Laminin | PA1-16730 | Invitrogen | Rabbit  Polyclonal | 1:100 |
| Lumican | MA5-34828 | Invitrogen | Rabbit  Monoclonal | 1:100 |
| Decorin | PA5-27370 | Invitrogen | Rabbit  Polyclonal | 1:100 |
| Alexa fluor 488 | A21206 | Invitrogen | Donkey anti-rabbit  Polyclonal | 1:1000 |
